# Oxidative Stress and Oocyte Cryopreservation: Recent Advances in Mitigation Strategies Involving Antioxidants

**DOI:** 10.3390/cells11223573

**Published:** 2022-11-11

**Authors:** Beijia Cao, Jianpeng Qin, Bo Pan, Izhar Hyder Qazi, Jiangfeng Ye, Yi Fang, Guangbin Zhou

**Affiliations:** 1 Key Laboratory of Livestock and Poultry Multi-Omics, Ministry of Agriculture and Rural Affairs, and Farm Animal Genetic Resources Exploration and Innovation Key Laboratory of Sichuan Province, College of Animal Science and Technology (Institute of Animal Genetics and Breeding), Sichuan Agricultural University, Chengdu 611130, China; 2 Department of Veterinary Anatomy, Histology, and Embryology, Shaheed Benazir Bhutto University of Veterinary and Animal Sciences, Sakrand 67210, Pakistan; 3 Jilin Provincial Key Laboratory of Grassland Farming, Northeast Institute of Geography and Agroecology, Chinese Academy of Sciences, Changchun 130102, China

**Keywords:** antioxidants, cryopreservation, mitophagy, oocyte, oxidative stress, ROS

## Abstract

Oocyte cryopreservation is widely used in assisted-reproductive technology and animal production. However, cryopreservation not only induces a massive accumulation of reactive oxygen species (ROS) in oocytes, but also leads to oxidative-stress-inflicted damage to mitochondria and the endoplasmic reticulum. These stresses lead to damage to the spindle, DNA, proteins, and lipids, ultimately reducing the developmental potential of oocytes both in vitro and in vivo. Although oocytes can mitigate oxidative stress via intrinsic antioxidant systems, the formation of ribonucleoprotein granules, mitophagy, and the cryopreservation-inflicted oxidative damage cannot be completely eliminated. Therefore, exogenous antioxidants such as melatonin and resveratrol are widely used in oocyte cryopreservation to reduce oxidative damage through direct or indirect scavenging of ROS. In this review, we discuss analysis of various oxidative stresses induced by oocyte cryopreservation, the impact of antioxidants against oxidative damage, and their underlying mechanisms. We hope that this literature review can provide a reference for improving the efficiency of oocyte cryopreservation.

## 1. Introduction

More than three hundred years ago, preserving cells or embryos below zero degrees was almost a fantasy. In 1949, the discovery that sperm could survive in glycerol at low temperatures [1] sparked the rapid development of cell cryopreservation. In 1972, the cryopreservation of mouse embryos was successfully completed [2]. Five years later, mature mouse oocyte cryopreservation was also achieved successfully, and live offspring were obtained following in vitro fertilization (IVF) embryo transfer in mice [3]. In 1985, with the invention of vitrification technology, researchers were able to avoid the formation of ice crystals during cryopreservation, and the damage to oocytes and embryos caused due to low temperatures was greatly reduced [4]. Both traditional slow freezing [2] and vitrification [4] have been successfully applied for the cryopreservation of rat [5], rabbit [6], and primate [7] oocytes, preserving a large number of excellent germplasm resources. Similarly, cryopreservation has remained at the center of attention for clinicians, and efforts have been put into reshaping the landscape of fertility treatment in humans [8]. For instance, there has been an increasing demand for preserving fertility opportunities for women who are suffering from ovarian disease, or for those who plan to have late marriage and childbearing [9].

However, despite advances in our understanding of cryopreservation protocols, there has been a lot of talk about improving the developmental rate of oocytes after cryopreservation which is still lower compared to their control/fresh counterparts. For example, recently it has been reported that the in vitro maturation rate of vitrified-warmed mouse germinal vesicle (GV) oocytes was decreased from 84% to 68% and the blastocyst formation rate was decreased from 27.8% to 20% [10]. Moreover, the rate of blastocyst formation of vitrified-warmed bovine M II oocytes was nearly 10% lower than that of fresh oocytes [11]. Mechanical damage of oocytes by ice crystals [12,13], toxic effects of cryoprotective agents [14], oxidative stress induced by excessive accumulation of intracellular reactive oxygen species (ROS) [15], and osmotic stress induced by low temperatures [14] can all lead to a reduced developmental potential of the oocytes. Among these, oxidative stress triggered by excessive ROS levels has attracted much attention and become an important issue. In this review article, we discuss this focused area and shed light on recent insights related to the impacts and mechanisms of exogenous antioxidants in oocyte cryopreservation.

## 2. ROS and Oxidative Stress

**Figure 1:**
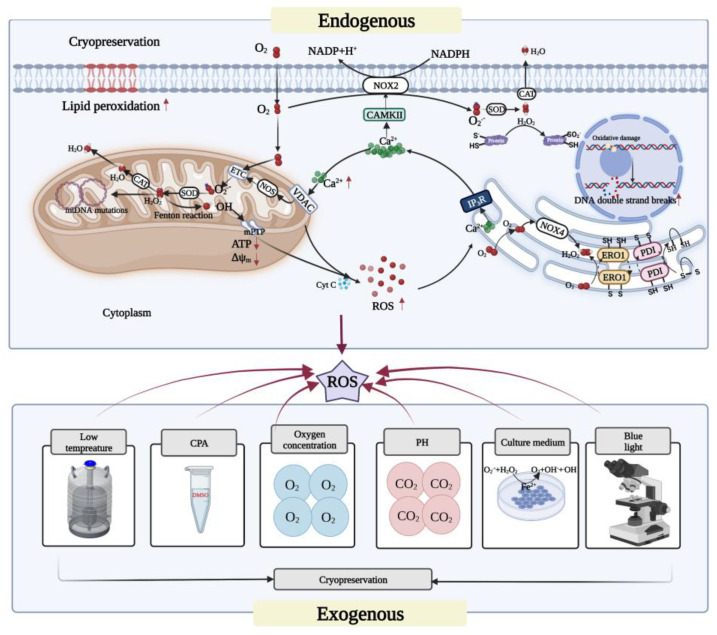
The sources of ROS in oocyte cryopreservation and its effects on oocytes. The exogenous ROS during oocyte cryopreservation mainly come from the environment and the reagents used in the experimental procedures. Mitochondria are the main sites for the generation of endogenous ROS, and some oxidases can also generate ROS. The ROS generated in the respiratory chain are released in large quantities through mPTP, which can enhance protein misfolding and trigger ER stress. Meanwhile, a large amount of ROS can also be generated in the ER. In addition, the ER releases large amounts of Ca^2+^ into the cytoplasm, which generates ROS, in part through NOX. Others are absorbed by the anion channel protein VDAC in mitochondria, causing Ca^2+^ overload, which further increases the levels of ROS produced by mitochondria, decreases ATP and mitochondrial membrane potential, and causes oxidative damage to cellular lipids, proteins, and DNA. ↑ and ↓, respectively, indicate a significant increase or decrease. Created with BioRender.com (accessed on 19 October 2022).

There are three main forms of ROS: superoxide radical (O_2_^•−^), hydrogen peroxide (H_2_O_2_), and hydroxyl radical (•OH). Among these, O_2_^•−^ has a short half-life and generally does not react with most biomolecules in the cytoplasmic water environment [16]. However, superoxide dismutase (SOD) can oxidize O_2_^•−^ disproportionately to H_2_O_2_. H_2_O_2_ has a longer half-life than O_2_^•−^, reacts selectively with other biomolecules, and can bind to enzymes such as catalase and glutathione to produce water and oxygen [17]. However, the •OH generated by H_2_O_2_ through the Fenton reaction is a reactive oxygen that readily reacts with everything around it, oxidizing amino acids and destroying protein conformation [15]. Compounding the problem, there is currently no known enzyme that can detoxify •OH. Consequently, •OH is the most toxic form of these three reactive oxygen species [15,18]. These and other aspects relevant specifically to oocyte cryopreservation are schematically summarized in Figure 1.

It is well established that under physiological conditions, ROS can act as a signaling molecule to control crucial life processes. However, when production of ROS exceeds the limit that can be eliminated by antioxidant defense mechanisms, intracellular redox homeostasis is disrupted and oxidative stress rises, resulting in intracellular oxidative DNA damage [19], an increase in DNA breaks [20], oxidative protein damage [21], and lipid peroxidation [22]. Therefore, it is necessary to reduce oxidative stress to preserve cell quality.

## 3. Sources of ROS in Oocyte Cryopreservation

ROS in oocyte cryopreservation can originate from both intracellular production and external environmental factors (see Figure 1). During oxidative metabolism, the intracellular source of ROS is mainly from the by-products produced by mitochondrial complexes I and III in the respiratory chain. ROS are also produced by oxidative enzymatic reactions of NADPH oxidase (NOX) [17] and nitric oxide synthase (NOS) located on the cell membrane and in the cytoplasm. Furthermore, during endoplasmic reticulum stress (ERS), the endoplasmic reticulum can also produce ROS through oxidative enzymes such as NADPH oxidase 4 (NOX4).

Extracellular sources of ROS include cryoprotective agents such as dimethyl sulfoxide (DMSO), which promotes the release of large amounts of calcium ions (Ca^2+^) from the endoplasmic reticulum into the cytoplasm, and then the uptake of more Ca^2+^ by the mitochondria, inducing more ROS production [23]. In turn, the extreme stimulus of low temperatures can exacerbate the production of ROS in the presence of DMSO [23]. Given that oocyte cryopreservation is performed in vitro, certain light in the experimental environment, such as blue light (400–500 nm), can cause oxidative damage to cells by generating H_2_O_2_ and altering enzymes in the respiratory chain [24,25], with microscopy accounting for 95% of the harmful radiation [26].Other types of ambient light such as UVB (290–320 nm) can induce DNA base oxidation and DNA strand breaking [27]. It has been reported that the exposure to visible light over five minutes can significantly increase the ROS level in mouse embryos [28].The pH gradient during in vitro culture as well as the oxygen partial pressure can also affect ROS levels. Higher pH values and oxygen concentrations lead to an increase in oxidase activity, inducing an increase in intracellular O_2_^•−^ levels [29]. In addition, metal ions such as Fe^2+^ and Cu^2+^ in the in vitro media can also induce ROS production through the Fenton reaction and the Haber–Weiss reaction, and Fe can also act directly on lipids to amplify peroxidative damage [29].

In small and medium-sized animal species, handling and preservation of ovaries is one of the crucial steps to ensure an efficient oocyte-cryopreservation protocol. Therefore, it is necessary to strictly control the transport time and temperature while ovaries are transported from slaughterhouse to the laboratory. For instance, it has been reported that ROS can rapidly generate O_2_^•−^ in sheep ovarian cells [30], which has been attributed to accumulation of xanthine oxidase and hypoxanthine during ischemia, resulting in an increased level of ROS levels and reaching a peak at 3 h [31]. The preservation of ovaries at too high (above 40 °C) or too low (below 20 °C) temperatures can cause heat stress and or cold shock in bovine oocytes, leading to increased intracellular ROS and affecting the quality of oocytes and subsequent embryo development [32,33]. In addition, drastic temperature changes during oocyte cryopreservation are also responsible for the increased levels of ROS. Heat stress during thawing of oocytes can also lead to mitochondrial dysfunction and changes in Ca^2+^ influx via oxidative stress, thus reducing the in vitro maturation rate of vitrified goat oocytes [34].

## 4. Cryopreservation-Induced Oxidative Stress in Oocytes

### 4.1. Mitochondrial Oxidative Stress

It is established that oxidative stress induced by cryopreservation can cause mitochondrial Ca^2+^ overload, and the mitochondrial permeability transition pore (mPTP) will continue to open, leading to further increase in Δψ and ATP dissipation [35] and ROS levels. Mitochondria have both inner and outer membranes, and the electron transport chain (ETC) in the inner mitochondrial membrane consists of mitochondrial complexes I to IV, along which electrons reduce oxygen molecules to water (see Figure 1). However, even under the physiological conditions, about 1% of the O_2_ is reduced to O_2_^•−^ due to electron leakage from the ETC [36] and then rapidly generates H_2_O_2_ [37]. H_2_O_2_ can continuously produce •OH through the Fenton reaction [38] and thus becomes the main site of intracellular ROS generation (see Figure 1). A large amount of ROS accumulation can induce severe oxidative stress in the mitochondria, thereby impairing the normal physiological activities of cells.

Oxidative stress leads to mitochondrial lipid and protein peroxidation, as well as mutations in mitochondrial DNA (mtDNA). Unlike other phospholipids, cardiolipin on the inner mitochondrial membrane is highly susceptible to ROS attack due to its proximity to ROS-producing sites [39]. Cytochrome C is released into the cytosol from cardiolipin. When it binds to apoptotic protein activator 1 (Apaf-1) and caspase-9 precursor, the caspase cascade pathway [39] can be activated and apoptosis promoted. As a key intermediate of the tricarboxylic acid cycle in mitochondria, cis-aconitate, its active region of Fe-S protein is blunted and inactivated once subjected to oxidative stress, leading to the formation of large amounts of Fe^2+^ and H_2_O_2_, causing oxidative metabolic dysfunction and decreased ATP production [40]. Given the fact that the mtDNA is closer to the site of free radical production, lacks a histone protection and noncoding region, it (mtDNA) is more susceptible to oxidative damage than the nuclear DNA [41]. Any expression or structural alteration of the mtDNA-encoded respiratory chain protein complex can, in turn, cause defects in the electron transport chain by inhibiting the electron flow of the complexes I, III, and IV, enhancing the electron transport to O_2_, and thereby generating more ROS and less ATP [42]. 

Cryopreservation can induce severe mitochondrial oxidative stress in oocytes. Oocyte maturation is accompanied by increased ATP and mitochondrial membrane potential (MMP, Δψ) [43,44]. In contrast, under low-temperature stimulation, Δψ is decreased while ROS levels are increased in porcine [45], mouse [46], and human oocytes [47], eventually leading to a decrease in oocyte maturation rate. In addition, oxidative stress induced by cryopreservation is believed to cause mitochondrial Ca^2+^ overload and opening of mitochondrial permeability transition pores (mPTPs), leading to Δψ and ATP dissipation and a further increase in ROS levels [35]. Moreover, cytochrome C and pro-apoptotic factors are also released [48], which accelerates oocytes apoptosis.

### 4.2. Endoplasmic-Reticulum-Related Oxidative Stress

When oxidative stress is established in cells, the redox homeostasis within the endoplasmic reticulum is disrupted and the correct folding of proteins is impaired, which in turn triggers endoplasmic reticulum stress (ERS). The occurrence of ERS is accompanied by the production of large amounts of ROS, further exacerbating the oxidative stress. The main enzymes mediating the ROS production in the endoplasmic reticulum during ERS are protein disulfide isomerase (PDI), endoplasmic reticulum oxidoreductin-1 (ERO-1), and NADPH oxidase 4 (reduced nicotinamide adenine). PDI and ERO-1 can transfer electrons to molecular oxygen and generate H_2_O_2_ through the flavin adenine dinucleotide (FAD)-dependent reaction [49], while Nox4 uses the NADH or NADPH as an electron donor to produce superoxide anion [50].

The increase in ROS levels induced by low temperatures can force oocytes to undergo persistent ERS [51], which disrupts intracellular Ca^2+^ homeostasis and the redox state. Upon ERS activation, Ca^2+^ stored in the ER is rapidly released into the cytoplasm through IP3R; some of which promotes NOX2-dependent ROS generation by CAMKII [52]. However, others are taken up by the mitochondria, leading to a Ca^2+^ overload that promotes the respiratory chain to generate more ROS through NOS. Meanwhile, mPTP is continuously open and accelerates apoptosis [53]. In contrast, inhibition of ERS reduces apoptosis and improves the developmental potential of vitrified-warmed mouse oocytes in vitro [51].

## 5. Antioxidant Mechanisms in Oocytes

### 5.1. Ribonucleoprotein Particles Can Reduce Oxidative Damage and Protect Cells

Mammalian ribonucleoprotein particles (RNPs) are granules composed of RNA and protein, including stress granules (SGs) and processing bodies (PBs) [54]. SGs are membrane-less dense messenger ribonucleoprotein (mRNP) particles formed in the cytoplasm, containing polyadenylated mRNAs forced to suspend translation due to the stress [55], small ribosomal subunits (40S), translation initiation factor PABP, and RNA binding protein TIA/1R. Similarly, several non-RNA-binding proteins such as signal transduction proteins are also contained in SGs [56]; however, RNAs whose translation is induced by stress are not included in the SGs [57].

Under physiological conditions, PBs contain a large number of mRNAs, protein complexes related to 5′-3′ mRNA degradation such as XRN1 and DCP1 [58], and proteins related to RNA translation inhibition such as GW182 [59]. PBs become larger and more numerous when they are subjected to stress [60,61,62], whereas SGs can only be formed by stress induction. It is precisely due to the formation of the SGs and PBs that cells are able to selectively store mRNAs so that they can recover quickly from various stressful environments.

SGs appear to dock with PBs under oxidative stress. Oxidative damage to RNA results in the formation of 8-hydroxyguanine (8-OHG) at the RNA guanine base. Y box-binding protein 1 (YB-1), which specifically recognizes and binds 8-OHG, is present in PBs [63]. YB-1 is transferred from PBs to SGs under certain types of stress. During stress recovery, the SGs can act as a “sorting” domain for mRNA [63], where mRNA without oxidative damage may be recycled and returned to the polysome to restart protein synthesis. Conversely, oxidized mRNA is transferred to the adjacent PBs for catabolism. Furthermore, SGs are resistant to oxidative damage and their antioxidant activity is regulated by their core components, the GTPase-activating protein SH3 domain-binding protein 1 (G3BP1) and the ubiquitin-specific protease 10 (USP10) [64].

**Figure 2:**
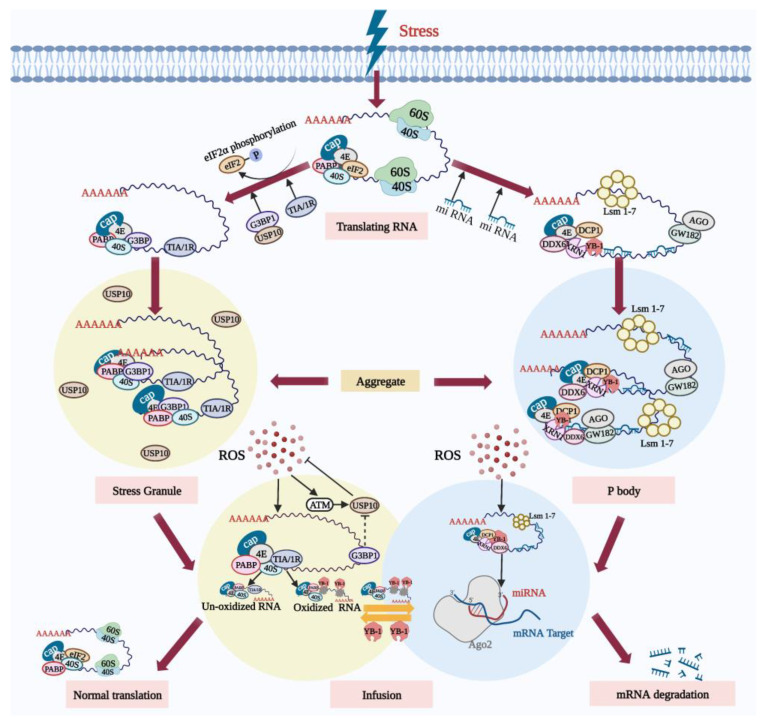
SGs and PBs actively participate in the degradation of oxidized RNA during oxidative stress. Following oxidative stress, the translation of some mRNA in cells is inhibited. On the one hand, the stress-induced phosphorylation of eIF2α inhibits translation initiation, and assembly of the stalled 48S initiation complex promotes the formation and aggregation of SGs. A large amount of RNA that has paused translation is sequestered in the SGs. SGs’ core component G3BP1 relieves the inhibition of USP10. ROS activates ATM and promotes the phosphorylation of USP10, triggering the antioxidant response. On the other hand, the stress-induced PBs mediate the RNA degradation. SGs and PBs dock, and RNA without oxidative damage can be re-translated under stress conditions. However, oxidized RNA is recognized by the YB-1 protein and transferred to PBs for degradation. Created with BioRender.com (accessed on 19 October 2022).

Under physiological conditions, excessive G3BP1 is able to inhibit the antioxidant activity of USP10 to maintain ROS homeostasis. However, upon exposure to external stress such as high concentrations of hydrogen peroxide [65], the inhibition of USP10 by G3BP1 is abrogated and USP10 is induced to phosphorylate by ataxia-telangiectasia-mutated protein (ATM) or activated by ATM-phosphorylated protein, thereby reducing ROS production and apoptosis [64]. Therefore, after cells are subjected to oxidative stress, SGs, on the one hand, dock with P-bodies. On the other hand, as a component of the antioxidant mechanisms, SGs are actively involved in the process of reducing oxidative damage of cells. All these processes relevant to SGs and PBs are depicted in Figure 2.

### 5.2. Mitophagy Can Reduce Oocyte Apoptosis Induced by Oxidative Stress 

**Figure 3:**
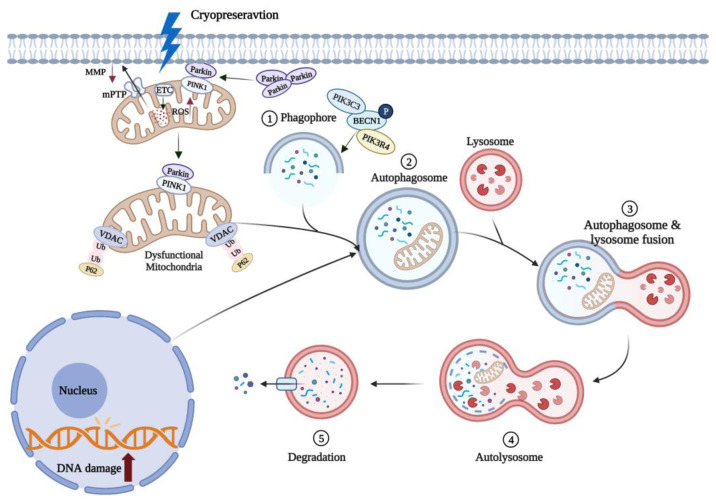
Mitophagy can remove oxidized mitochondria in oocytes. Cryopreservation causes oxidative stress in oocytes and rapid depolarization of MMP, which promotes Parkin entry into damaged mitochondria. Parkin induces polyubiquitination of VDAC in mitochondria, and ubiquitinated VDAC recruits p62 into mitochondria to induce mitophagy. These damaged mitochondria are phagocytosed by autophagic vesicles to form autophagosomes. Mature autophagosomes fuse with lysosomes to form autolysosomes, and the contained mitochondria are subsequently degraded. ↑ and ↓, respectively, indicate a significant increase or decrease. Created with BioRender.com (accessed on 19 October 2022).

Mitophagy is a special autophagic process that selectively removes excessive or damaged mitochondria (see Figure 3). MMP loss heralds the onset of mitophagy and induces accumulation of PTEN-inducible kinase1 (PINK1) in the mitochondrial outer membrane, which regulates Parkin recruitment into the damaged mitochondria [66]. Damaged mitochondria can be selectively phagocytosed by autophagic vesicles to form double-membrane autophagosomes and then fuse into lysosomes to be degraded by hydrolytic enzymes [67]. Parkin is an E3 ubiquitous ligase that promotes mitochondrial autophagy in damaged mitochondria. Mitophagy plays an important role in regulating intracellular mitochondrial number and maintaining mitochondrial function.

It has been experimentally demonstrated that cryopreservation (low-temperatures) can promote mitophagy [68]. The expression level of Beclin1 (BECN1), a key regulator of autophagy in oocytes, was significantly increased following mouse oocyte cryopreservation [68], while the accumulation of P62, a characteristic substrate of autophagy, was decreased [69]. Recently, it has been shown that mitophagy can improve the survival of vitrified-warmed oocytes by eliminating oxidation-damaged mitochondria (see Figure 3). Lately, it has been demonstrated that vitrified-thawed porcine MII oocytes can regulate cell activity through PINK/Parkin-mediated mitophagy [70]. When mitophagy was inhibited with chloroquine (CQ), mitochondrial dysfunction and oxidative damage was increased in vitrified porcine oocytes, reducing the in vitro oocyte developmental potential [71]. Following knockdown of autophagy-related genes in yeast, damaged mitochondria that are not cleared will continue to generate excessive ROS, compromising cell survival [72,73]. It is interesting to note that mitophagy does not always protect the vitrified oocytes, but excessive mitophagy can also lead to a decreased mouse oocyte developmental potential in vitro [74]. This evidence collectively highlights the fact that the mitophagy-mediated protection of vitrified-warmed oocytes is so far limited, therefore, further mechanistic studies are required to improve our understanding of this fascinating phenomenon.

## 6. Oocyte Cryopreservation and Antioxidants

### 6.1. Endogenous Antioxidants Resist Oxidative Damage Induced by Oocyte Cryopreservation

There are three major enzymatic antioxidants in cells, namely superoxide dismutase (SOD), catalase (CAT), and glutathione peroxidase (GPX) [75]. SOD can provide an initial detoxification of O_2_^•−^, and its products are then further detoxified to water by CAT or GPX. The upregulation of *SOD* gene expression levels in mouse and porcine oocytes after vitrification [76,77] facilitates the scavenging of cryopreservation-induced ROS, but the decrease in GSH levels [78] exacerbates the oxidative stress in cells to the extent that 50 IU/mL SOD needs to be added to the freezing solution to scavenge the excessive ROS and to improve fertilization and survival of mouse oocytes [79]. In vitrified-warmed sheep oocytes, it was observed that the CAT activity was significantly higher compared to their control counterparts during in vitro culture [80]. Similarly, the addition of catalase during vitrification of goat ovarian tissue was effective in reducing freezing-induced accumulation of ROS [81].

In addition, glutathione (GSH), cysteine (CYS), and cysteamine (CSH) are key endogenous non-enzymatic antioxidants in oocytes. GSH synthesized in oocytes can regulate the sulfur–oxygen reduction state of cells [82], and promote cytoplasmic maturation. GSH can protect against the oxidative-stress-inflicted damage to the morphology and function of the spindle during in vitro maturation of bovine oocytes [83], and improve the development potential of sheep embryos [84]. Cysteine, a small mercaptan that can enhance the synthesis of GSH, can be added to bovine oocyte maturation medium to reduce oxidative damage, potentially by increasing the level of GSH [85]. Cysteamine can also directly scavenge •OH [86], contributing to maintenance of redox status and high GSH/GSSG ratio in the oocytes. However, after vitrification, the intracellular synthesis of GSH was significantly reduced, and the addition of CYS or CSH also failed to significantly improve the blastocyst formation rate of vitrified-warmed bovine oocytes after IVF [87]. 

It, therefore, appears that the exogenous antioxidants are beneficial in reducing cryo-oxidative damage, particularly due to the fact that endogenous enzymatic antioxidants alone are unable to mitigate cryopreservation-inflicted ROS.

### 6.2. Exogenous Non-Enzymatic Antioxidants Protect Oocytes from Oxidative Damage Induced by Cryopreservation

To reduce the cryopreservation-inflicted oxidative damage to oocytes, non-enzymatic antioxidants are usually applied in the vitrification, warming, and/or culture media to mitigate the oxidative stress. Numerous antioxidants including melatonin [46,88,89], resveratrol [90,91,92], L-carnitine [93,94], quercetin [95], vitamin E [96,97], astaxanthin [98], proline [99], and coenzyme Q10 [100] have been demonstrated to have beneficial impact on oocyte maturation and development. The results of the previous studies provide encouraging evidence that the oxidative damage in vitrified-warmed oocytes can be reduced/mitigated, and their in vitro developmental potential can be greatly improved. In Table 1, we have summarized the fascinating results of relevant studies involving different antioxidants, including those from our group.

Recently, it has been shown that mitochondria-targeted antioxidants are more effective compared to the traditional antioxidants. For instance, mitoquinone (MitoQ), which consists of a triphenylphosphate positive cation is covalently attached to the benzoquinone portion of coenzyme Q10 in the respiratory chain complex through a ten-carbon aliphatic chain, can selectively scavenge excessive mitochondrial ROS [102] to maintain ROS homeostasis in mitochondria [103]. MitoQ can also protect the integrity of mitochondrial membranes in vitrified-warmed oocytes. It has been reported that the addition of 2 × 10^−8^ mol/L MitoQ to the freezing solution significantly enhanced the mitochondrial membrane potential and cell survival in vitrified-warmed mouse MII-stage oocytes [101]. However, single administration of targeted antioxidants or autophagy activators such as rapamycin to reduce oxidative stress has limited effects, so dual-targeted therapies that can simultaneously modulate antioxidant signaling pathways and autophagy are being explored [104].

There is an increasing need to upgrade the existing antioxidants to amplify their antioxidant properties. It is promising that the search for forming efficient remedial strategies involving potent antioxidants is also at the center of attention of reproductive biology researchers and clinicians. For instance, curcumin is not only used in clinical applications for its anti-cancer effects, but also has attracted great attention from researchers around the world for its antioxidant and oxygen-radical-scavenging effects [105,106]. Its chemical structure was modified to obtain a new antioxidant molecule, acetyl zingerone, whose ability to reduce H_2_O_2_ and •OH is 17.7 and 39.6 times greater than that of the traditional antioxidant vitamin E, respectively [107]. Not only this but it has a stable structure that can withstand ultraviolet light and maintain its high activity, while vitamin E basically drops to practically zero [107]. Therefore, the exploitation of acetyl zingerone is expected to improve the efficiency of oocyte cryopreservation.

It is important to note that the impact of supplementation of exogenous antioxidants on oocyte development has been shown to be concentration-dependent and varies between species [108,109,110]. Even within the same species, antioxidants can render different effects based on the developmental stages of the oocytes at which they are used [108,109,110]. Intriguingly, there is evidence that, instead of having beneficial effects, high concentrations of antioxidants can also disrupt cell development by interfering with intracellular antioxidant mechanisms and converting oxidative stress into reductive stress [111,112], leading to further damage. There is an evidence that antioxidants can sometimes render more beneficial effects when used in combination or in different chemical forms/presentations than those used alone. For instance, co-encapsulation of melatonin and resveratrol in solid lipid nano-carriers (SLNS) may provide more effective antioxidant synergy and can significantly reduce ROS levels in vitrified-warmed mouse GV-stage oocytes [113]. However, not all antioxidants are more effective when used in combination. This notion is supported by evidence that the single administration of catalase had better clonogenic parameters compared to its combination with alginose [114].

## 7. Mechanism of Exogenous Non-Enzymatic Antioxidants and Their Implication in Reducing Oxidative Damage Following Oocyte Cryopreservation

### 7.1. Melatonin Reduces Oxidative Damage in Vitrified-Warmed Oocytes by Activating Antioxidant Signaling Pathways and Maintaining Organelle Morphology

**Figure 4:**
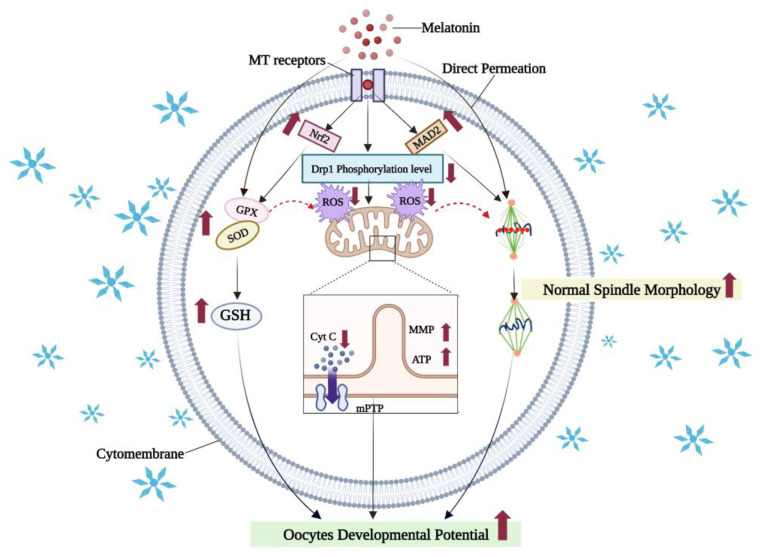
The mechanism of melatonin in reducing oxidative damages in vitrified oocytes. Melatonin can enter oocytes through receptors, act on the Nrf2 antioxidant signaling pathway, and enhance the expression of antioxidant enzymes, protect normal morphology and function of organelles such as mitochondria and the spindle, and ensure the correct segregation of meiotic chromosomes. Melatonin can also directly penetrate into cells to scavenge free radicals, reduce intracellular ROS, and improve the development potential of cells in vitro. Created with BioRender.com (accessed on 19 October 2022).

Melatonin is a natural endogenous indole hormone produced by the mammalian pineal gland [115]. It is also present in the liver [116] and ovaries [117]. Melatonin and its metabolites are powerful antioxidants [118,119], which can reduce oxidative stress by scavenging reactive oxygen species directly or indirectly [120]. Since melatonin is both lipid- and water-soluble [121], it can easily cross the cell membrane to transfer hydrogen and electrons to directly scavenge free radicals [122], reduce cellular ROS levels [123,124], and enhance the in vitro maturation rate of vitrified-warmed oocytes and the IVF blastocyst rate [118,125,126]. Moreover, melatonin may also act on antioxidant signaling pathways through its receptors (melatonin receptors, MTRs) to indirectly enhance glutathione peroxidase (GPX) activity [127], or promote the expression of the related antioxidant protein Nrf2 to reduce oxidative stress and improve in vitro maturation of vitrified-warmed mouse oocytes [89]. Some of the molecular mechanistic bases through which melatonin exerts its beneficial effects during oocyte cryopreservation are schematically summarized in the Figure 4.

Melatonin can also maintain normal morphology and function of vitrified-warmed oocyte organelles through MTRs [88,128]. Disturbances in spindle assembly and spindle assembly checkpoint (SAC)-related components caused by oocytes cryopreservation can lead to chromosomal segregation errors, which in turn increase the number of aneuploid embryos and cause miscarriages or congenital diseases [129]. It has been reported that 10^−7^ mol/L melatonin can improve spindle morphology through MTRs, regulate the levels of MAD2 protein, an important component of the SAC, and subsequently improve the in vitro developmental potential of vitrified-warmed mouse GV-stage oocytes [88]. In addition, oocyte cryopreservation can lead to increased phosphorylation levels of kinesin-related protein 1 (Drp1), while 10^−7^ mol/L melatonin was able maintain mitochondrial fission/fusion homeostasis via MTRs that reduce Drp1 phosphorylation levels, protecting mitochondrial function, reducing the release of pro-apoptotic genes and cytochrome C, and improving the survival rate of vitrified-warmed mouse GV-stage oocytes [130].

### 7.2. Resveratrol Can Reduce Oxidative Damage in Vitrified-Warmed Oocytes by Activating SIRT1 to Mediate Mitochondrial Function

Resveratrol is a natural non-flavonoid polyphenolic organic compound widely found in plants such as grapes, thuja, and peanuts. The phenolic hydroxyl group of resveratrol is highly redox-prone and can bind to reactive oxygen radicals [131] and activate antioxidant enzymes such as CAT and SOD [132], while inhibiting the activity of oxidative enzymes. Thereby, it can reduce intracellular ROS, increasing the maturation rate of vitrified-warmed cat oocytes in vitro [92] and promoting subsequent embryonic development [92,133].

**Figure 5:**
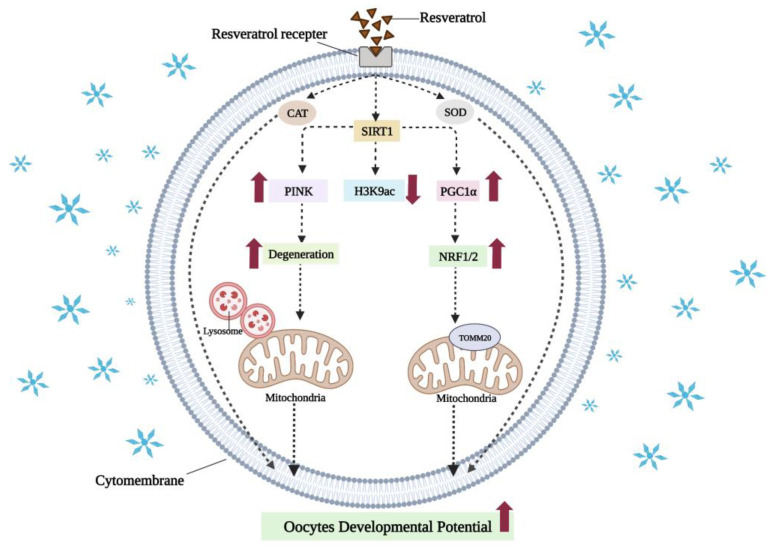
The mechanism of resveratrol in reducing oxidative damages of vitrified oocytes. Resveratrol can enter oocytes through receptors, activate the activity of antioxidant enzymes, and reduce oxidative stress caused by cryopreservation. It can also activate SIRT1 via receptors to deacetylate PGC1α, and activate NRF1 and NRF2 to enhance mitochondrial biosynthesis. Epigenetically, resveratrol can also reduce abnormal H3K9 acetylation levels via SIRT1. In addition, resveratrol can promote the degradation of damaged mitochondria, maintain the quality of mitochondria, and restore the developmental potential of oocytes. ↑ and ↓, indicate a significant increase or decrease, respectively. Created with BioRender.com (accessed on 19 October 2022).

Resveratrol is also a natural activator of the silencing regulatory protein 1 (sirtuin1, SIRT1), which deacetylates PPARγ coactivator-1α (PGC1α) and improves mitochondrial function by activating NRF1, NRF2 (see Figure 5), and downstream genes such as *TFAM* [134]. The addition of 10^−6^ mol/L resveratrol activated SIRT1 and enhanced the expression of mitochondrial-synthesis-related genes and increased the mtDNA copy numbers. The redox state of oocytes was improved, oxidative damage to oocytes caused by cryopreservation was reduced, and the maturation rate of vitrified-warmed porcine oocytes was significantly increased [69]. It has been reported that resveratrol can also improve the developmental potential of vitrified-warmed mouse oocytes in vitro by activating SIRT1 to regulate the epigenetic modification mechanism [135]. SIRT1 is an important histone deacetylase. There is evidence that, following vitrification, H3K9 acetylation was significantly increased and DNA methylation levels were significantly reduced in oocytes. It is encouraging to note that these abnormal changes were rescued by addition of 2 × 10^−6^ mol/L resveratrol in mice [135].

### 7.3. Other Antioxidants Can Also Eliminate Reactive Oxygen Species to Reduce Oxidative Damage Directly or Iindirectly in Vitrified-Warmed Oocytes

N-acetyl-cysteine (NAC) contains mercaptans that react with free-radical side chains to directly scavenge excessive intracellular ROS. As a precursor of cysteine, the more important indirect antioxidant effect of NAC is achieved by enhancing intracellular GSH stores. Upon entry into the cell, NAC is deacetylated by the action of N-deacetylase and produces acetyl and L-cysteine [136]. Under the action of glutamate cysteine ligase (GCL), L-cysteine binds to L-glutamic acid (GLU) and subsequently synthesizes GSH with the combined action of GSH synthetase (GS) and L-glycine (GLI), thus effectively reducing intracellular ROS levels [136]. Recently, it has been shown that the addition of 10^−3^ mol/L NAC after cryopreservation of mouse MII-stage oocytes can effectively improve the blastocyst formation [137]. Furthermore, 1.5 × 10^−3^ mol/L NAC was shown to significantly reduce ROS, improve mitochondrial distribution, and increase the rate of oocyte development in vitrified-warmed mouse GV-stage oocytes [138].

Quercetin, as a natural flavonoid antioxidant, can not only directly scavenge free radicals, but also inhibit the activity of oxidase [139] and lipid peroxidation [140]. It can also activate the Nrf2-ARE antioxidant pathway to improve the activities of downstream related antioxidant enzymes such as SOD and GPX to protect cells from oxidative damage [141,142]. Very recently, it has been reported that addition of 5 × 10^−6^ mol/L quercetin was effective in improving the maturation and reducing the apoptosis of vitrified-warmed mouse GV-stage oocytes in vitro, as well as increasing the oocyte cleavage rate and blastocyst formation rate after in vitro fertilization [95].

Vitamin E is a lipid-soluble vitamin that is abundant in cell membranes, and its antioxidant function is mainly to inhibit lipid peroxidation of membrane phospholipids or lipoproteins [143], which can lead to impaired cell-membrane function and permeability, and ultimately cell apoptosis. In addition to its impact on protection against polyunsaturated fatty acids in membranes, vitamin E is involved in keeping iron and other metal elements in a reduced state [97]. A study observed that 3 × 10^−4^ mol/L vitamin E can reduce intracellular ROS levels and significantly increase the IVF blastocyst rate in vitrified-warmed bovine MII-stage oocytes [96].

## 8. Conclusions and Future Perspectives

After years of research, it is now established that oocyte cryopreservation can induce mitochondrial oxidative stress, mitochondrial lipid and protein peroxidation, mtDNA mutation, and decreased mitochondrial ATP and membrane potential. Meanwhile, a variety of apoptotic factors are released, which then cause oocyte apoptosis. Oxidative stress can also be induced in the endoplasmic reticulum, which releases large amounts of Ca^2+^ into the cytoplasm, which are taken up by the mitochondria, leading to mitochondrial Ca^2+^ overload and further accelerating oocyte apoptosis.

There is evidence that oocytes can scavenge oxidized mitochondria, to some extent, by mitophagy or degrade (or compartmentalize) oxidized RNA by ribonucleoprotein particles. However, it is fascinating to note that these intrinsic antioxidant mechanisms of oocytes fail to be effective under the vigor of ultra-low temperatures and vitrification, and that, quantitatively, they are not enough to completely eliminate the oxidative-stress-inflicted damage in oocytes following cryopreservation. Even with the addition of exogenous antioxidants or targeted antioxidants, the extent of oxidative damage could not be largely restored to pre-vitrification levels, and the developmental potential of vitrified-warmed oocytes remained lower compared to their fresh counterparts. Therefore, the quest to improve outcomes following cryopreservation of oocytes is compelling the scientific community to exploit additional antioxidants remedies. As such, there is need to further investigate the underlying mechanistic basis, and come up with reasonable solutions to effectively reduce cryopreservation-inflicted oxidative damage to oocytes and improve their developmental competence.

## Figures and Tables

**Table 1:** The effects of exogenous non-enzymatic antioxidants on oocyte cryopreservation.

Components	Concentration	Oocyte Types	Effects	References
Melatonin	10^−7^ mol/L	Mouse GV-stage oocytes	(↓) ROS levels, spindle damage	[89]
	10^−9^ mol/L	Human MII-stage oocytes	(↑) Blastocyst formation rate, Oocyte maturation rate, MMP, ATP	[46]
Resveratrol	1.5 × 10^−3^ mol/L	Cat GV-stage oocytes	(↑) Oocyte maturation rate, cleavage rate, embryo developmental ratio	[92]
	5 × 10^−6^ mol/L	Cat COC complexes	(↓) ROS levels (↑) GSH levels	[91]
	2 × 10^−6^ mol/L	Porcine MII-stage oocytes	(↓) Cell apoptosis	[90]
Quercetin	5 × 10^−6^ mol/L	Mouse GV-stage oocytes	(↑) Oocyte maturation rate, embryo developmental ratio	[95]
Vitamin E	3 × 10^−4^ mol/L	Bovine MII-stage oocytes	(↑) Blastocyst formation rate	[96]
	10^−4^ mol/L	Mouse MII-stage oocytes	(↑) Oocyte morphology and ultrastructure	[97]
Astaxanthin	2.5 × 10^−6^ mol/L	Porcine GV-stage oocytes	(↑) GSH levels, lysosomal activity (↓) ROS levels, cathepsin B activity	[98]
Proline	2 mol/L	Mouse MII-stage oocytes	(↑) Spindle and mitochondrial function	[99]
Coenzyme Q10	5 × 10^−5^ mol/L	Bovine COC complexes	(↑) Cell survival after vitrification (↑) Migration of cortical granule	[100]
L-carnitine	0.8 × 10^−3^ mol/L	Porcine COC complexes	(↑) SOD1 gene expression	[93]
	0.6 g/mL	Mouse COC complexes	(↑) GSH levels	[94]
MitoQ	2 × 10^−8^ mol/L	Mouse MII-stage oocytes	(↑) MMP, cell survival	[101]

↑ and ↓ indicate a significant increase or decrease, respectively.

## Data Availability

Not applicable.
